# Preprocedural computed tomography angiography aortic root geometry and 1-year left ventricular reverse remodeling after transcatheter aortic valve replacement

**DOI:** 10.3389/fcvm.2026.1924035

**Published:** 2026-09-07

**Authors:** Xiaoting Li, Daisong Jiang, Jian Liu, Changdong Zhang, Xiaoke Shang, Nianguo Dong, Ye Yang, Jian Yang, Haibo Zhang, Min Jin, Qing Zhou, Chunshui Liang, Yuan Zhao, Buqing Ni, Yongfeng Shao, Zhong Wu

**Affiliations:** 1 Department of Cardiovascular Surgery, West China Hospital, Sichuan University, Chengdu, China; 2 Department of Cardiovascular Surgery, Guangdong Cardiovascular Institute, Guangdong Provincial People’s Hospital, Guangdong Academy of Medical Sciences, Southern Medical University, Guangzhou, China; 3 Department of Cardiovascular Surgery, Union Hospital, Tongji Medical College, Huazhong University of Science and Technology, Wuhan, China; 4 Department of Cardiovascular Surgery, Zhongshan Hospital, Fudan University, Shanghai, China; 5 Department of Cardiovascular Surgery, Xijing Hospital, Air Force Medical University, Xi’an, China; 6 Department of Cardiac Surgery, Beijing Anzhen Hospital, Capital Medical University, Beijing, China; 7 Department of Cardiac Surgery, Nanjing Drum Tower Hospital, The Affiliated Hospital of Nanjing University Medical School, Nanjing, China; 8 Department of Cardiovascular Surgery, Xinqiao Hospital, Army Medical University (Third Military Medical University), Chongqing, China; 9 Department of Cardiovascular Surgery, The Second Xiangya Hospital, Central South University, Changsha, China; 10 Department of Cardiovascular Surgery, Jiangsu Province Hospital, The First Affiliated Hospital of Nanjing Medical University, Nanjing, China

**Keywords:** aortic root anatomy, aortic stenosis, computed tomography, left ventricular reverse remodeling, TAVR—transcatheter aortic valve replacement

## Abstract

**Introduction and objectives:**

Left ventricular reverse remodeling (LVRR) after transcatheter aortic valve replacement (TAVR) is associated with outcomes, but the relevance of aortic-root geometry remains uncertain. We examined whether the annular eccentricity index (EI) and sinus of Valsalva height on computed tomography angiography (CTA) were associated with 1-year LVRR.

**Methods:**

This retrospective secondary analysis included 115 patients with severe aortic stenosis from an 11-center cohort who underwent successful TAVR and survived to undergo evaluable preprocedural CTA and 1-year echocardiography. EI was calculated as the difference between maximum and minimum annular diameters divided by the maximum diameter. Sinus height was the mean perpendicular distance from the annular plane to the sinus tip across the three sinuses. LVRR was defined as an increase in left ventricular ejection fraction of at least 5 percentage points or a decrease in indexed left ventricular end-diastolic volume of at least 15%. A fixed multivariable logistic model adjusted for baseline ventricular function and clinical covariates; Firth bias-reduced logistic regression was used for primary inference because the smaller outcome group contained 37 patients, with conventional maximum-likelihood estimation as a sensitivity analysis.

**Results:**

LVRR occurred in 78 patients (67.8%). In the primary Firth analysis, each 1-standard deviation (SD) decrease in EI was associated with higher odds of LVRR (adjusted odds ratio, 2.40; 95% confidence interval, 1.52–3.92; *P* < 0.001), as was each 1-SD increase in sinus height (adjusted odds ratio, 1.84; 95% confidence interval, 1.23–2.74; *P* = 0.003). Conventional maximum-likelihood estimates were directionally consistent. Model performance was lower after bootstrap optimism correction (area under the curve, 0.77 vs. 0.81 apparent).

**Conclusions:**

Among patients who survived to undergo evaluable 1-year echocardiography, a lower EI and greater sinus height were associated with LVRR after TAVR. These hypothesis-generating associations require external validation before clinical application.

## Introduction

Transcatheter aortic valve replacement (TAVR) is the standard of care for symptomatic severe aortic stenosis (AS) across surgical risk strata (1). Mechanical relief of pressure overload triggers left ventricular reverse remodeling (LVRR) in most patients, yet between one-third and one-half fail to meet conventional LVRR thresholds at 1 year despite a technically successful procedure (2, 3). Because the magnitude of post-TAVR LVRR tracks closely with subsequent survival and heart-failure events (2, 4), preprocedural identification of patients with reduced reverse-remodeling potential carries direct relevance to surveillance intensity and adjunctive medical therapy. The present study is a secondary analysis of the registered multicenter Xcor cohort (5).

Most existing predictive markers center on myocardial substrate—late gadolinium enhancement on cardiac magnetic resonance, extracellular volume fraction, or strain echocardiography (4, 6)—or on postprocedural hemodynamics. Preprocedural valve–root anatomy, by contrast, has received comparatively little attention. The aortic root is a three-dimensional structure relevant to transcatheter heart valve (THV) deployment and postimplantation flow (7, 8). Two descriptors readily available on routine pre-TAVR computed tomography angiography (CTA) have plausible, but untested, links to recovery. The annular eccentricity index (EI) quantifies departure from circularity and may relate to THV expansion symmetry and residual valve loading (9). Sinus of Valsalva height characterizes supravalvular root geometry and may influence sinus flow after THV deployment (10). These potential pathways were not directly evaluated in the present study.

We examined whether EI and sinus height, modeled as continuous variables, were associated with 1-year LVRR after adjustment for a fixed covariate set. Alternative outcome definitions, covariate sets, and valve-morphology subgroups were evaluated as supportive analyses.

## Methods

### Study design and population

This was a secondary retrospective analysis of prospectively collected data from a registered multicenter Xcor study that enrolled consecutive patients undergoing TAVR for native severe AS at 11 high-volume centers between May 2022 and May 2023. The present analysis was conditional on successful TAVR, survival to follow-up, diagnostic ECG-gated preprocedural CTA, and evaluable transthoracic echocardiography at baseline and 1 year. The permitted 1-year echocardiographic assessment window was 10–14 months after TAVR; the observed median timing was 12.1 months (interquartile range, 11.8–12.6 months). Mutually exclusive exclusions were failure to meet procedural-success criteria (*n* = 3), death within 30 days after TAVR (*n* = 5), death after 30 days but before the 1-year assessment (*n* = 1), loss to follow-up (*n* = 1), non-diagnostic preprocedural CTA (*n* = 2), prior cardiac surgery (*n* = 1), more than mild mitral or aortic regurgitation at baseline (*n* = 1), or non-diagnostic 1-year echocardiography (*n* = 1). The study followed the STROBE recommendations (11) and Declaration of Helsinki of the World Medical Association and was approved by the Ethics Committee on Clinical Trial, West China Hospital of Sichuan University [Approval No.: 2022 Clinical Trial (Device) Review No. (17); approval date: 23 February 2022]; the requirement for individual informed consent was waived for a retrospective analysis of deidentified data.

### TAVR procedure

All patients underwent transapical TAVR using the self-expanding Xcor system (Saint Medical Technology, Nanjing, China), which is designed exclusively for transapical delivery. Valve sizing followed the manufacturer's recommendations based on CTA-derived annular dimensions. Procedural success was defined according to VARC-3 criteria.

### CTA measurements and reproducibility

**Figure 1:**
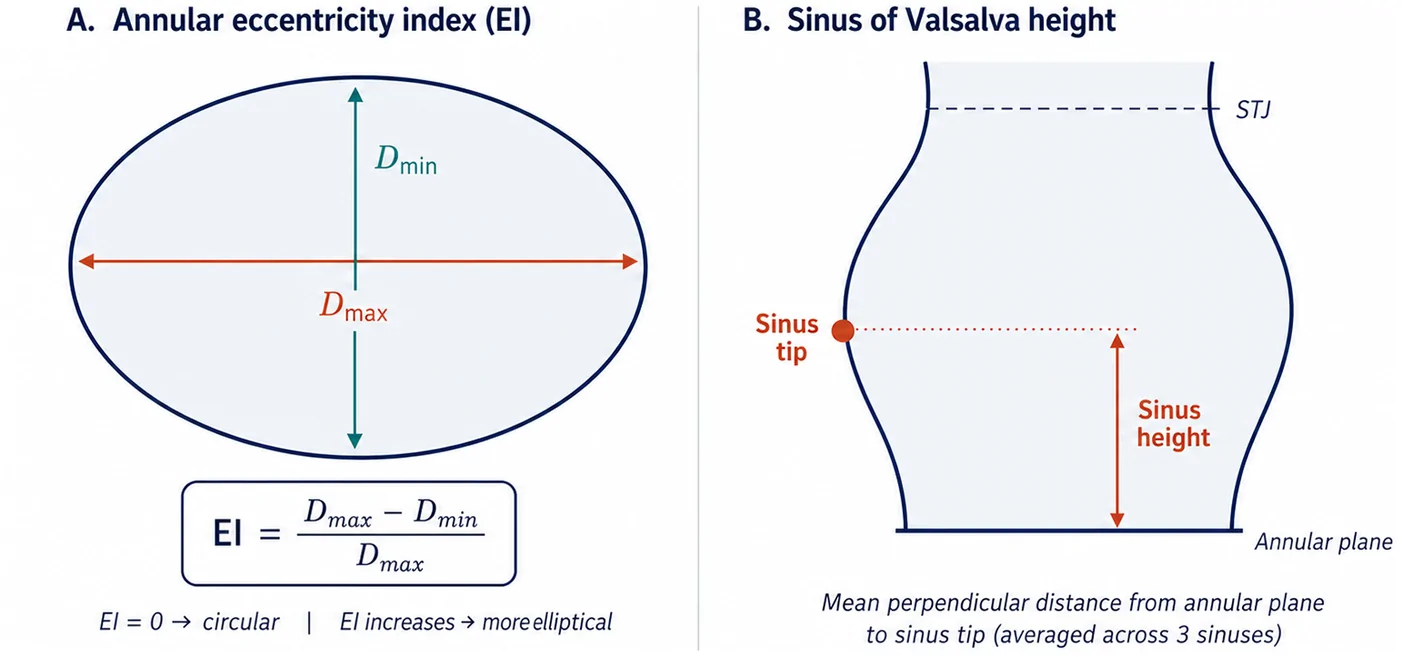
CTA-derived aortic root geometric parameters. **(A)** Annular eccentricity index (EI) at the virtual basal annular plane: EI = (*D*_max_−*D*_min_)/*D*_max_. EI = 0 denotes a circular annulus; rising values indicate increasing ellipticity. **(B)** Sinus of Valsalva height: mean perpendicular distance from the annular plane to the sinus tip, averaged across the three sinuses. STJ, sinotubular junction.

All CTA datasets were analyzed on a dedicated TAVR-planning workstation (CVPILOT/TAVIMercy, Nanjing, China) by two readers independently and blinded to outcomes; readings differing by more than 5% were adjudicated by consensus. Definitions are illustrated in Figure 1. EI was calculated at the virtual basal annular plane as (*D*_max_−*D*_min_)/*D*_max_, where *D*_max_ and *D*_min_ are the maximum and minimum annular diameters. Sinus of Valsalva height was the mean perpendicular distance from the annular plane to the sinus tip, averaged across the three sinuses. In a 30-patient random subset (26% of the cohort), interobserver reproducibility was excellent: EI intraclass correlation coefficient (ICC) 0.91 (95% CI 0.83–0.96; coefficient of variation 8.4%); sinus height ICC 0.94 (0.88–0.97; coefficient of variation 5.1%).

### Echocardiographic outcomes

Left ventricular ejection fraction (LVEF) was measured by the biplane Simpson method and indexed left ventricular end-diastolic volume (LVEDVI) by indexed end-diastolic volume on standardized protocols. The primary outcome was 1-year LVRR, defined as a ≥5-percentage-point increase in LVEF *or* a ≥15% decrease in LVEDVI (“OR-criterion”), consistent with prior LVRR literature (2, 3). To explore the dose dependence of geometry on the magnitude of remodeling, we additionally classified patients into three prespecified ordinal LVRR strata: *none* (neither criterion met), *partial* (one of the two criteria met), and *complete* (both criteria met). The strict definition (“AND-criterion,” complete LVRR only) and continuous ΔLVEF and ΔLVEDVI were prespecified as sensitivity outcomes.

### Statistical analysis

Continuous variables are summarized as mean ± standard deviation or median (interquartile range) according to distribution; categorical variables as *n* (%). Group comparisons were performed using the Student *t*-test, Mann–Whitney *U*-test, and *χ*^2^ or Fisher’s exact test as appropriate. Normality was assessed using the Shapiro–Wilk test.

**Figure 2:**
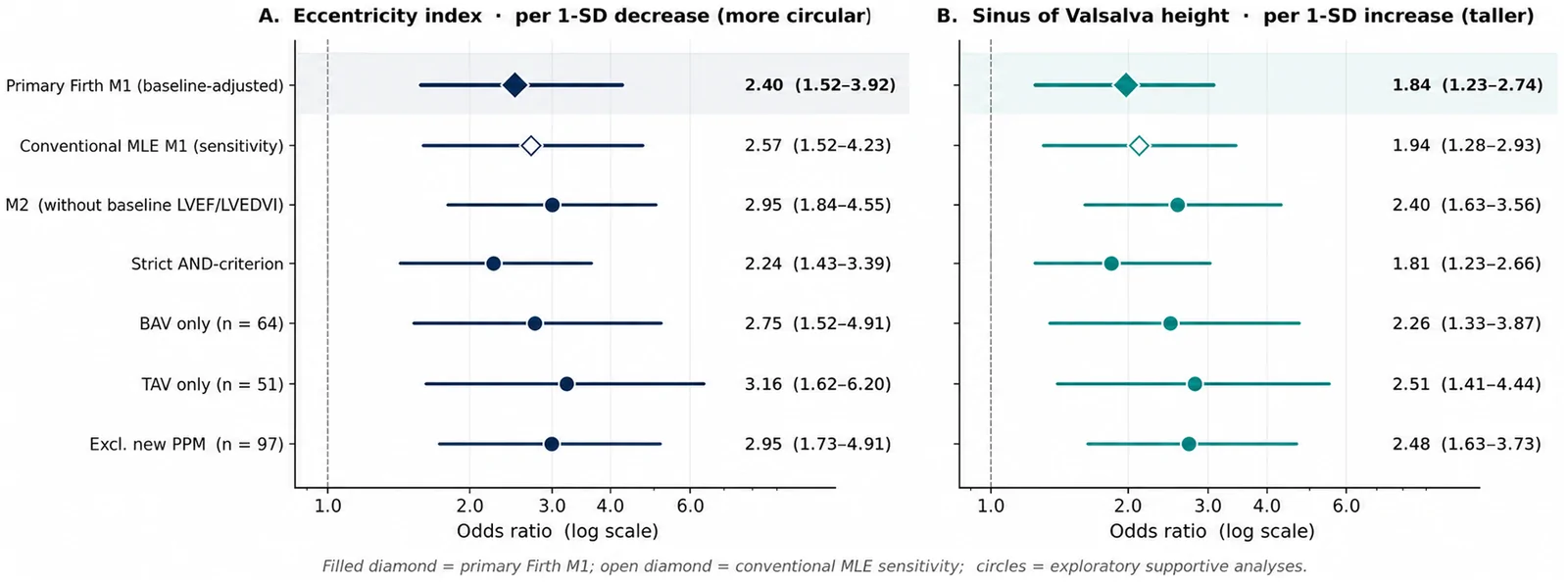
Primary and supportive effect estimates across model specifications. Standardized odds ratios (95% CI) for LVRR are shown in the clinically favorable direction for **(A)** annular eccentricity index per 1-SD decrease and **(B)** sinus of Valsalva height per 1-SD increase. The filled diamond is the primary Firth M1 estimate, the open diamond is the conventional maximum-likelihood M1 sensitivity estimate, and the circles are exploratory supportive estimates. The vertical dashed line denotes an odds ratio of 1. Primary Firth M1 estimates were 2.40 (95% CI, 1.52–3.92) for EI and 1.84 (95% CI, 1.23–2.74) for sinus height. The figure is descriptive and should not be interpreted as independent confirmation or proof of robustness. CI, confidence interval; EI, eccentricity index; LVRR, left ventricular reverse remodeling; MLE, maximum-likelihood estimation; PPM, permanent pacemaker; SD, standard deviation.

The fixed primary multivariable model included EI and sinus height as continuous predictors and adjusted for baseline LVEF, baseline LVEDVI, age, sex, valve morphology (bicuspid vs. tricuspid), Society of Thoracic Surgeons Predicted Risk of Mortality (STS-PROM), and left ventricular mass indexed to body surface area (LV mass index) (designated “M1”). This exact specification was used throughout the text, Table 1, Figures 2, and the Central Illustration. Variance inflation factors did not exceed 5 for any predictor.

**Table 1:** Multivariable association of CTA geometry with 1-year LVRR.

Specification	EI per 0.01-unit increase OR (95% CI); *P*	Sinus height per 1 mm OR (95% CI); *P*
Primary firth M1 (baseline-adjusted)	0.86 (0.79–0.93); <0.001	1.32 (1.10–1.58); 0.003
Conventional maximum-likelihood M1 (sensitivity analysis)	0.85 (0.78–0.93); <0.001	1.35 (1.12–1.63); 0.002
M2 (without baseline LVEF, LVEDVI)	0.83 (0.77–0.90); <0.001	1.49 (1.25–1.78); <.001
Strict AND-criterion	0.87 (0.81–0.94); 0.001	1.31 (1.10–1.56); 0.003
BAV subgroup (*n* = 64)	0.84 (0.76–0.93); <0.001	1.45 (1.14–1.85); 0.003
TAV subgroup (*n* = 51)	0.82 (0.73–0.92); <0.001	1.52 (1.17–1.97); 0.002
Excluding new PPM (*n* = 97)	0.83 (0.76–0.91); <0.001	1.51 (1.25–1.82); <0.001
Continuous outcomes (linear regression)		
ΔLVEF, percentage points (*β*; SE; *P*)	−0.38 (0.12); 0.002	+0.52 (0.19); 0.007
ΔLVEDVI, mL/m^2^ (*β*; SE; *P*)	+0.44 (0.14); 0.002	−0.65 (0.22); 0.004

Highlighted row = primary firth specification; conventional maximum-likelihood M1 and all other rows are sensitivity or exploratory supportive analyses. M1 covariates: age, sex, baseline LVEF, baseline LVEDVI, valve morphology, STS-PROM, and LV mass index. M2 covariates: age, sex, valve morphology, STS-PROM, and LV mass index. BAV by EI interaction *P* = 0.71; BAV by sinus-height interaction *P* = 0.64.

BAV, bicuspid aortic valve; CI, confidence interval; EI, eccentricity index; OR, odds ratio; PPM, permanent pacemaker; TAV, tricuspid aortic valve; other abbreviations as in Table 1.

**Central Illustration:**
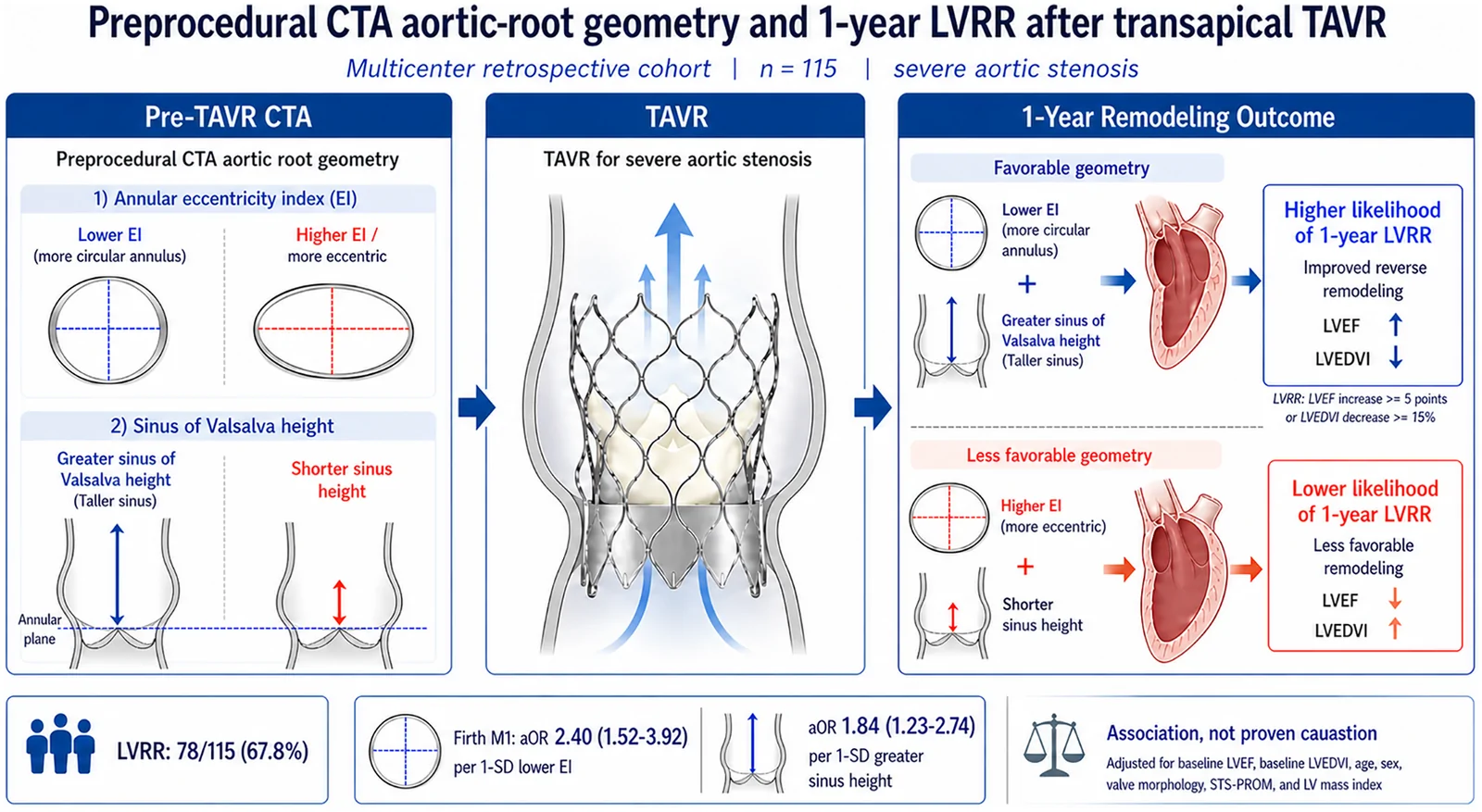
Preprocedural aortic-root geometry and 1-year left ventricular reverse remodeling after transapical TAVR. A lower annular eccentricity index and greater sinus of Valsalva height on preprocedural CTA were associated with higher odds of 1-year LVRR among patients who survived to undergo evaluable 1-year echocardiography. Primary Firth M1 adjusted for baseline LVEF, baseline LVEDVI, age, sex, valve morphology, STS-PROM, and LV mass index. Neutral connectors depict the observed association and do not imply a causal pathway through THV expansion, postprocedural hemodynamics, or sinus flow. EI, eccentricity index; LVEDVI, indexed left ventricular end-diastolic volume; LVEF, left ventricular ejection fraction; LVRR, left ventricular reverse remodeling; STS-PROM, Society of Thoracic Surgeons Predicted Risk of Mortality; TAVR, transcatheter aortic valve replacement; THV, transcatheter heart valve.

Six supportive analyses were performed, each substituting one element of the primary specification: (i) a model without baseline echocardiographic covariates (M2; age, sex, valve morphology, STS-PROM, LV mass index); (ii) the strict AND-criterion outcome; (iii) multivariable linear regression of continuous ΔLVEF and ΔLVEDVI; (iv) bicuspid (BAV) and tricuspid (TAV) subgroups, with multiplicative interaction terms; (v) exclusion of patients receiving a new permanent pacemaker (PPM) within 30 days; and (vi) ordinal trend across the no/partial/complete LVRR strata. Supportive, subgroup, and interaction analyses were considered exploratory because of limited statistical power.

With 37 patients in the smaller outcome category and nine model parameters (approximately 4.1 smaller-category events per parameter), conventional maximum-likelihood estimates were at risk of overfitting (12). Firth bias-reduced logistic regression was therefore used for primary inference, with conventional maximum-likelihood estimation retained as a sensitivity analysis (13). No variables were removed on the basis of observed significance. The Firth models used profile penalized-likelihood inference. Missing data (<3% across variables) were handled by multiple imputation (*m* = 20) (14). Internal validation used 2,000 bootstrap resamples of the complete modeling procedure; apparent and optimism-corrected area under the receiver operating characteristic curve (AUC), calibration slope, and calibration intercept were estimated from intercept-corrected Firth-predicted probabilities (15, 16). Analyses were performed in R 4.2.1 (R Foundation for Statistical Computing, Vienna, Austria) using two-sided *P* < 0.05.

## Results

### Cohort characteristics

**Figure 3:**
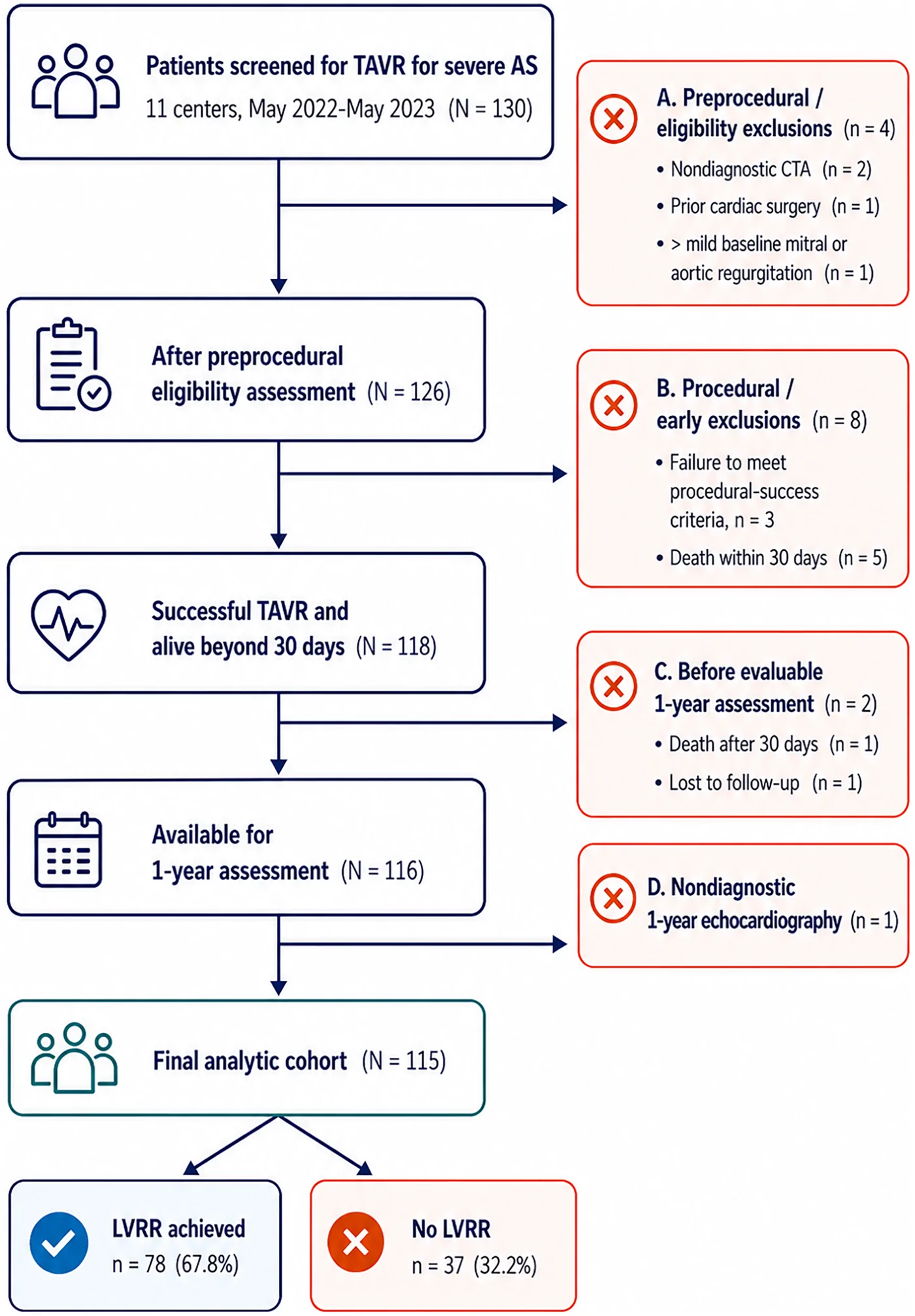
Study flow diagram. Of the 130 patients screened across 11 centers between May 2022 and May 2023, 115 were included in the final analytic cohort and 15 were excluded in mutually exclusive chronological stages: preprocedural or eligibility exclusions (*n* = 4: non-diagnostic CTA, *n* = 2; prior cardiac surgery, *n* = 1; more than mild baseline mitral or aortic regurgitation, *n* = 1); procedural or early exclusions (*n* = 8: failure to meet procedural-success criteria, *n* = 3; death within 30 days, *n* = 5); exclusions before the 1-year assessment (*n* = 2: death after 30 days but before the 1-year assessment, *n* = 1; loss to follow-up, *n* = 1); and non-diagnostic 1-year echocardiography (*n* = 1). LVRR was achieved in 78 patients (67.8%); 37 patients (32.2%) did not achieve LVRR. The analytic cohort was conditional on procedural success, survival, and evaluable 1-year echocardiography. CTA, computed tomography angiography; LVRR, left ventricular reverse remodeling.

Of the 130 patients screened, 115 met the eligibility criteria for this secondary analysis and were included in the final analytic cohort (Figure 3); 15 patients were excluded. The mutually exclusive exclusions were failure to meet procedural-success criteria (*n* = 3), death within 30 days after TAVR (*n* = 5), death after 30 days but before the 1-year assessment (*n* = 1), loss to follow-up (*n* = 1), non-diagnostic preprocedural CTA (*n* = 2), prior cardiac surgery (*n* = 1), more than mild mitral or aortic regurgitation at baseline (*n* = 1), and non-diagnostic 1-year echocardiography (*n* = 1). Among the included 115 patients, the mean age was 70.8 ± 4.2 years, 61 (53.0%) were males, and 64 (55.7%) had bicuspid valve anatomy. This high bicuspid proportion is consistent with reports from some Chinese TAVR cohorts (17) and should be considered when assessing transportability to cohorts consisting predominantly of patients with tricuspid anatomy. LVRR (OR-criterion) was achieved in 78 patients (67.8%); the strict AND-criterion (complete LVRR) was met in 48 (41.7%).

Baseline characteristics by LVRR status are summarized in Table 2. Patients who achieved LVRR had lower baseline LVEF (57.2 ± 12.6% vs. 62.5 ± 10.6%; *P* = 0.02), larger LVEDVI (88.8 ± 15.1 vs. 76.8 ± 14.2 mL/m^2^; *P* < 0.001), and a higher LV mass index (133.4 ± 35.1 vs. 118.2 ± 27.5 g/m^2^; *P* = 0.01). On CTA, patients with LVRR had a markedly more circular annulus (EI 0.209 ± 0.048 vs. 0.288 ± 0.039; *P* < 0.001) and taller sinuses (18.5 ± 2.3 vs. 16.6 ± 1.3 mm; *P* < 0.001).

**Table 2:** Baseline characteristics and 1-year echocardiographic changes.

Variable	Total (*N* = 115)	LVRR (*n* = 78)	No LVRR (*n* = 37)	*P*
Demographics and clinical				
Age (years)	70.8 ± 4.2	70.6 ± 3.5	71.2 ± 5.4	0.54
Male sex, *n* (%)	61 (53.0)	43 (55.1)	18 (48.6)	0.52
Body mass index (kg/m^2^)	22.7 ± 3.1	22.5 ± 3.3	23.1 ± 2.6	0.29
Bicuspid aortic valve, *n* (%)	64 (55.7)	47 (60.3)	17 (45.9)	0.15
Hypertension, *n* (%)	89 (77.4)	62 (79.5)	27 (73.0)	0.44
Diabetes mellitus, *n* (%)	28 (24.3)	18 (23.1)	10 (27.0)	0.64
Atrial fibrillation, *n* (%)	15 (13.0)	11 (14.1)	4 (10.8)	0.77
STS-PROM (%)	7.6 ± 2.7	7.5 ± 2.5	7.8 ± 3.0	0.60
Baseline echocardiography				
LVEF (%)	58.9 ± 12.2	57.2 ± 12.6	62.5 ± 10.6	0.02
LVEDVI (mL/m^2^)	84.9 ± 15.8	88.8 ± 15.1	76.8 ± 14.2	<0.001
LV mass index (g/m^2^)	128.5 ± 33.5	133.4 ± 35.1	118.2 ± 27.5	0.01
Aortic valve area, (cm^2^)	0.68 ± 0.20	0.67 ± 0.18	0.70 ± 0.24	0.50
Mean gradient (mmHg)	54.5 ± 22.7	56.3 ± 21.8	50.7 ± 24.4	0.24
Preprocedural CTA aortic root geometry				
Eccentricity index	0.235 ± 0.058	0.209 ± 0.048	0.288 ± 0.039	<0.001
Sinus of Valsalva height, mm	17.9 ± 2.2	18.5 ± 2.3	16.6 ± 1.3	<0.001
1-year echocardiographic changes				
ΔLVEF, percentage points	+3.1 ± 7.5	+7.3 ± 4.5	−5.7 ± 4.0	<0.001
ΔLVEDVI (mL/m^2^)	−12.2 ± 17.0	−21.6 ± 10.8	+7.7 ± 8.2	<0.001

Continuous variables: mean ± SD. Categorical: *n* (%).

LVEDVI, indexed left ventricular end-diastolic volume; LVEF, left ventricular ejection fraction; STS-PROM, Society of Thoracic Surgeons Predicted Risk of Mortality.

ΔLVEF and ΔLVEDVI denote 1-year minus baseline.

### Echocardiographic changes at 1 year

LVEF rose by +7.3 ± 4.5 percentage points among patients with LVRR and fell by −5.7 ± 4.0 points among those without; LVEDVI fell by 21.6 ± 10.8 mL/m^2^ in those with LVRR and rose by 7.7 ± 8.2 mL/m^2^ in those without (both *P* < 0.001; Table 2). Of the 78 patients meeting the OR-criterion, 48 (61.5%) met both criteria, 18 (23.1%) met the LVEF criterion only, and 12 (15.4%) the LVEDVI criterion only. Across the full cohort, 66 (57.4%) achieved a ≥5-point LVEF gain and 60 (52.2%) a ≥15% LVEDVI reduction.

### Primary association of CTA geometry with LVRR

In the fixed primary M1 model adjusted for baseline LVEF, baseline LVEDVI, age, sex, valve morphology, STS-PROM, and LV mass index, the Firth bias-reduced odds ratio was 0.86 per 0.01-unit increase in EI (95% CI, 0.79–0.93; *P* < 0.001) and 1.32 per 1-mm increase in sinus height (95% CI, 1.10–1.58; *P* = 0.003). Expressed in the clinically favorable direction, the corresponding standardized Firth odds ratios were 2.40 (95% CI, 1.52–3.92; *P* < 0.001) for each 1-SD decrease in EI and 1.84 (95% CI, 1.23–2.74; *P* = 0.003) for each 1-SD increase in sinus height. Conventional maximum-likelihood estimates were 0.85 (95% CI, 0.78–0.93; *P* < 0.001) per 0.01-unit increase in EI and 1.35 (95% CI, 1.12–1.63; *P* = 0.002) per 1-mm increase in sinus height.

**Figure 4:**
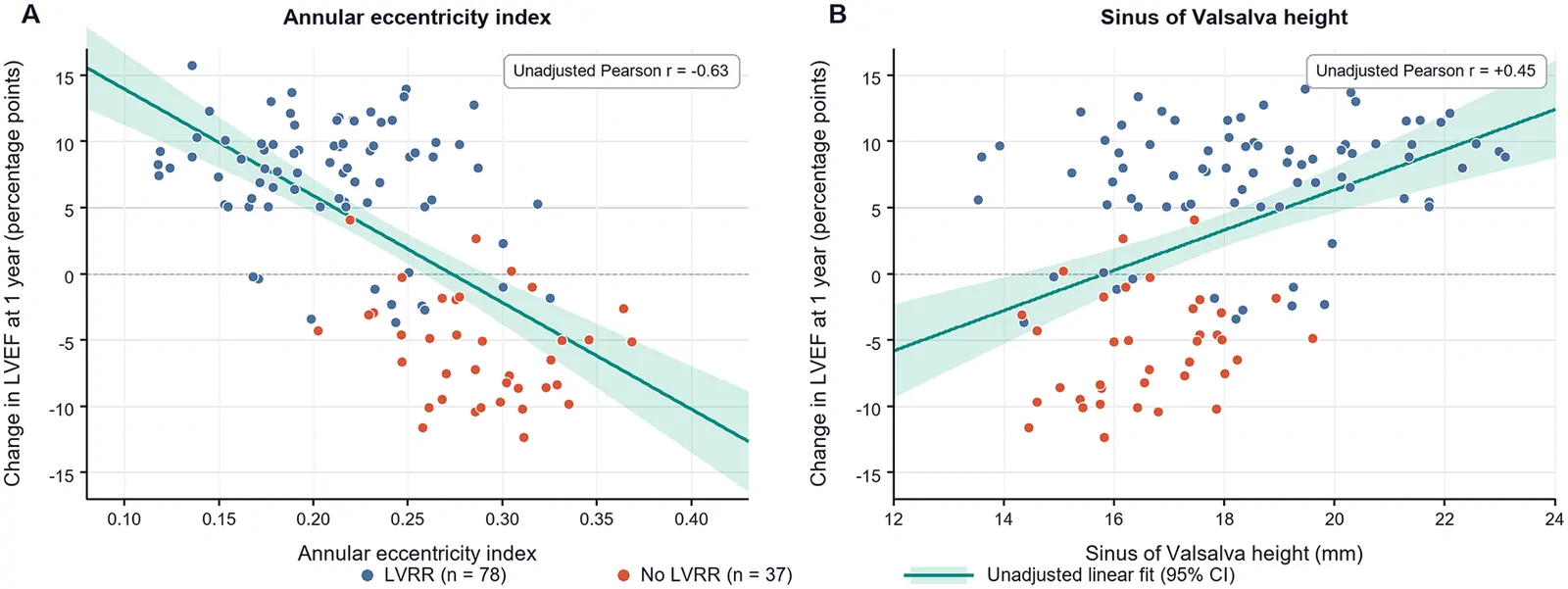
Unadjusted continuous associations of CTA geometry with 1-year change in LVEF. Each point represents one patient (blue, LVRR; coral, no LVRR). The solid lines are unadjusted ordinary least-squares fits, and the shaded bands are 95% confidence intervals. **(A)** Annular eccentricity index vs. change in LVEF. **(B)** Sinus of Valsalva height vs. change in LVEF. The displayed Pearson *r* values are exploratory and unadjusted. Adjusted beta coefficients are reported separately in the Results and Table 1 and are not displayed in the figure. CTA, computed tomography angiography; EI, eccentricity index; LVEF, left ventricular ejection fraction; LVRR, left ventricular reverse remodeling.

Figure 4 displays exploratory unadjusted patient-level associations of EI and sinus height with change in LVEF. The ordinary least-squares fitted lines, 95% confidence bands, and Pearson correlations were unadjusted: *r* = −0.63 for EI and *r* =  + 0.45 for sinus height. In supportive multivariable linear models reported separately in the text and Table 1, the adjusted coefficients were beta = −0.38 per 0.01-unit increase in EI (*P* = 0.002) and beta =  + 0.52 per 1-mm increase in sinus height (*P* = 0.007). A quartile analysis showed monotonic LVRR proportions (EI Q1 to Q4: 86%, 76%, 62%, 46%; sinus Q1 to Q4: 48%, 59%, 76%, 89%; both *P*-for-trend <0.001); these descriptive gradients do not establish a clinical threshold or causal dose–response relationship.

For the intercept-corrected Firth model, the apparent AUC was 0.81 (95% CI, 0.73–0.89). Across 2,000 bootstrap resamples, mean optimism was 0.04, yielding an optimism-corrected AUC of 0.77 (95% CI, 0.68–0.85), a calibration slope of 0.82, and a calibration intercept of −0.02. These internally validated performance estimates are exploratory and do not substitute for external validation.

### Graded LVRR analysis

Across the three supportive LVRR strata, both geometric descriptors showed monotonic gradients (Table 3). Patients with no LVRR had the highest EI (0.288 ± 0.039) and shortest sinuses (16.6 ± 1.3 mm); those with partial LVRR were intermediate (EI 0.225 ± 0.052; sinus 17.9 ± 2.0 mm); those with complete LVRR (both criteria) had the lowest EI (0.198 ± 0.043) and tallest sinuses (18.9 ± 2.4 mm). *P*-for-trend across the three strata was <0.001 for both descriptors. This descriptive pattern is consistent with a graded association but does not establish a causal dose–response relationship or a clinical threshold.

**Table 3:** CTA aortic root geometry across graded LVRR strata.

LVRR stratum	Criteria met	*n* (%)	EI	Sinus height (mm)
None	Neither	37 (32.2)	0.288 ± 0.039	16.6 ± 1.3
Partial	One criterion only	30 (26.1)	0.225 ± 0.052	17.9 ± 2.0
Complete	Both criteria	48 (41.7)	0.198 ± 0.043	18.9 ± 2.4
*P*-for-trend	–	–	<0.001	<0.001

Values are mean ± SD, unless otherwise indicated. LVRR strata defined by component criteria (LVEF increase ≥5 percentage points; LVEDVI decrease ≥15%).

EI, eccentricity index.

### Supportive analyses

In conventional maximum-likelihood supportive analyses, effect directions were consistent with those of the conventional M1 sensitivity model (Figure 2; Table 1). When baseline LVEF and LVEDVI were removed from the covariate set (M2), EI per 0.01-unit increase had an odds ratio of 0.83 (95% CI, 0.77–0.90; *P* < 0.001), while sinus height per 1 mm had an odds ratio of 1.49 (95% CI, 1.25–1.78; *P* < 0.001). Under the strict AND-criterion, the corresponding estimates were 0.87 (95% CI, 0.81–0.94; *P* = 0.001) for EI and 1.31 (95% CI, 1.10–1.56; *P* = 0.003) for sinus height. Supportive continuous-outcome regressions yielded associations with both ΔLVEF (EI beta = −0.38, *P* = 0.002; sinus beta =  + 0.52, *P* = 0.007) and ΔLVEDVI (EI beta =  + 0.44, *P* = 0.002; sinus beta = −0.65, *P* = 0.004). These analyses were exploratory and should not be interpreted as independent confirmation.

Stratified estimates were directionally concordant in BAV (EI OR, 0.84; 95% CI, 0.76–0.93; *P* < 0.001; sinus OR, 1.45; 95% CI, 1.14–1.85; *P* = 0.003) and TAV (EI OR, 0.82; 95% CI, 0.73–0.92; *P* < 0.001; sinus OR, 1.52; 95% CI, 1.17–1.97; *P* = 0.002); multiplicative interaction terms by valve morphology were not significant (EI × morphology *P* = 0.71; sinus × morphology *P* = 0.64), although power was limited. We note that interaction tests at this sample size are underpowered, and the absence of a significant interaction does not constitute proof of homogeneity. Excluding 18 patients with new PPM within 30 days left the effects essentially unchanged (EI OR, 0.83; 95% CI, 0.76–0.91; *P* < 0.001; sinus OR, 1.51; 95% CI, 1.25–1.82; *P* < 0.001).

## Discussion

In this multicenter analytic cohort of 115 patients who survived to undergo evaluable 1-year echocardiography after TAVR for severe AS, a lower annular eccentricity index and greater sinus of Valsalva height were associated with LVRR after adjustment for a fixed set of baseline ventricular and clinical covariates. In the primary Firth model, the odds ratio was 0.86 (95% CI, 0.79–0.93) per 0.01-unit increase in EI and 1.32 (95% CI, 1.10–1.58) per 1-mm increase in sinus height. Conventional maximum-likelihood estimates and supportive analyses were directionally consistent, but the low number of smaller-category events and the optimism observed during internal validation require cautious interpretation. Although the direction of association may be genuine, the magnitude of the observed effects may be inflated by the small sample size, selection of the analytic cohort, and model optimism and may attenuate in external validation. The findings identify hypothesis-generating anatomical associations rather than a validated prediction tool or causal mechanism.

### Biological context

The observed associations have plausible anatomical context, but the present design does not permit causal inference. Annular geometry may relate to THV expansion symmetry and residual valve loading (9, 18), while sinus height may characterize aspects of supravalvular flow geometry (10, 19). However, postprocedural transvalvular gradients, paravalvular regurgitation, effective orifice area, THV expansion symmetry, and sinus flow were not uniformly measured. These pathways therefore remain hypotheses derived from prior literature rather than mechanisms demonstrated in this cohort, and no mediation analysis was performed.

### Comparison with prior work

Most contemporary post-TAVR risk markers center on myocardial tissue properties—late gadolinium enhancement, extracellular volume fraction, or strain (4, 6)—rather than on root anatomy. Root geometry may provide complementary anatomical information, but the present study did not directly compare it with a complete set of myocardial substrate markers. The marked 55.7% bicuspid prevalence in our cohort warrants particular consideration because bicuspid aortic stenosis presents distinct anatomical challenges for TAVR (20). A bicuspid proportion of 48.5% was reported in the Chinese CARRY registry, whereas bicuspid anatomy accounted for 3.2% of procedures in a large United States TAVR registry (17, 21). These between-cohort differences may reflect geographic variation as well as differences in age, referral patterns, eligibility criteria, and treatment selection rather than ethnicity alone. Although exploratory subgroup estimates were directionally concordant in patients with BAV and TAV, the interaction tests were underpowered and the sample was insufficient to exclude clinically meaningful effect-size heterogeneity. Transportability to cohorts consisting predominantly of patients with tricuspid anatomy requires external evaluation.

### Clinical implications

EI and sinus height can be derived from routine pre-TAVR planning CTA without additional image acquisition or contrast. The observed graded patterns suggest that these variables may be evaluated as continuous anatomical descriptors rather than dichotomized at a data-derived threshold. However, incremental value beyond a clinical–echocardiographic model was not established, and neither operating thresholds nor clinical utility were evaluated. These findings should therefore be tested in prospectively defined external cohorts rather than used for current treatment or surveillance decisions.

### Limitations

First, postprocedural hemodynamic data—30-day residual mean gradient, paravalvular regurgitation grade, and effective orifice area—were not uniformly captured and were not analyzed. THV expansion symmetry and sinus flow were also not measured; the proposed mechanical pathway therefore remains hypothesis-generating. Second, only 37 patients were in the smaller outcome category for a fixed 9-parameter model. Firth estimation reduces small-sample and separation-related bias but cannot eliminate overfitting, model instability, or optimism; the internally validated performance remains exploratory, and external validation is absent. The strong unadjusted correlations may also be influenced by selection, residual confounding, mathematical coupling, and regression-to-the-mean because changes in baseline ventricular measures contribute to the outcome definition. Third, the retrospective analysis was conditional on procedural success, survival, and evaluable 1-year echocardiography; death and loss to follow-up can introduce selection bias, and the 10–14-month assessment window permits limited timing variability around the 1-year target. Fourth, the 55.7% bicuspid prevalence rate is consistent with those of some Chinese TAVR cohorts but, together with the underpowered interaction tests, limits generalizability to cohorts consisting predominantly of patients with tricuspid anatomy. Fifth, cardiac magnetic resonance fibrosis markers, B-type natriuretic peptide, troponin, and strain echocardiography were not uniformly available, preventing comparison with contemporary substrate markers. Sixth, all imaging used a single planning platform; cross-vendor reproducibility, particularly for EI (ICC 0.91), is a prerequisite for any future clinical deployment.

## Conclusions

Among patients who survived and underwent evaluable 1-year echocardiographic follow-up after transapical self-expanding TAVR, a lower annular eccentricity index and greater sinus of Valsalva height on preprocedural CTA were associated with a higher likelihood of left ventricular reverse remodeling. This survivor-conditioned, hypothesis-generating association requires external validation and direct evaluation of postprocedural hemodynamic mechanisms before clinical application.

## Data Availability

The raw data supporting the conclusions of this article will be made available by the authors, without undue reservation.
